# A Practical Roadmap for Clinical Translation of Metabolic Biomarkers: A Review

**DOI:** 10.3390/ijms27042030

**Published:** 2026-02-21

**Authors:** Kyung-Hee Kim, Maro Yoo, Min Yeong Choi, Byong Chul Yoo

**Affiliations:** 1 Department of Applied Chemistry, School of Science and Technology, Kookmin University, Seoul 02707, Republic of Korea; 2 Antibody Research Institute, Kookmin University, Seoul 02707, Republic of Korea; 3 Center for Liver and Pancreatobiliary Cancer, Hospital, National Cancer Center, Goyang 10408, Republic of Korea; 4 Division of Cancer Early Detection, National Cancer Control Institute, National Cancer Center, Goyang 10408, Republic of Korea; 5 Diagnostic Research Team, InnoBation Bio R&D Center, Seoul 03929, Republic of Korea

**Keywords:** metabolomics, lipidomics, biomarker translation, analytical validity, clinical validation, cohort heterogeneity, multi-metabolite panels

## Abstract

Metabolomics and lipidomics enable comprehensive profiling of metabolic states across diverse diseases and have generated a vast number of candidate biomarkers. Despite this progress, only a small fraction of metabolite-based biomarkers have achieved durable clinical translation. While this gap is often attributed to biological complexity or limited cohort size, increasing evidence suggests that failure more commonly reflects systematic misalignment between analytical measurement, biological interpretation, and clinical decision-making requirements. In this review, we argue that metabolites are not intrinsically unreliable biomarkers but are frequently overinterpreted as disease-specific indicators despite being highly context-dependent reporters of physiological state. We synthesize recurrent failure modes across the translational pipeline—including pre-analytical instability, ionization bias and semi-quantitative measurement, structural and annotation ambiguity, statistical overfitting, loss of disease specificity under systemic stress, and cohort-dependent performance collapse. Building on these insights, we propose a structured roadmap for the clinical translation of metabolite and lipid biomarkers. Rather than emphasizing further discovery, this framework prioritizes decision-oriented eligibility criteria encompassing pre-analytical robustness, analytical validity, molecular definition, biological interpretability, validation under real-world heterogeneity, and alignment with clinical utility and regulatory expectations. By reframing metabolic biomarkers as context-sensitive measurements embedded within clinical decision systems, this review provides practical guidance for investigators, clinicians, and regulators seeking to translate metabolomics and lipidomics into reliable tools for clinical practice.

## 1. Introduction: Systematic Attrition of Metabolic Biomarkers in Clinical Translation

**Figure 1:**
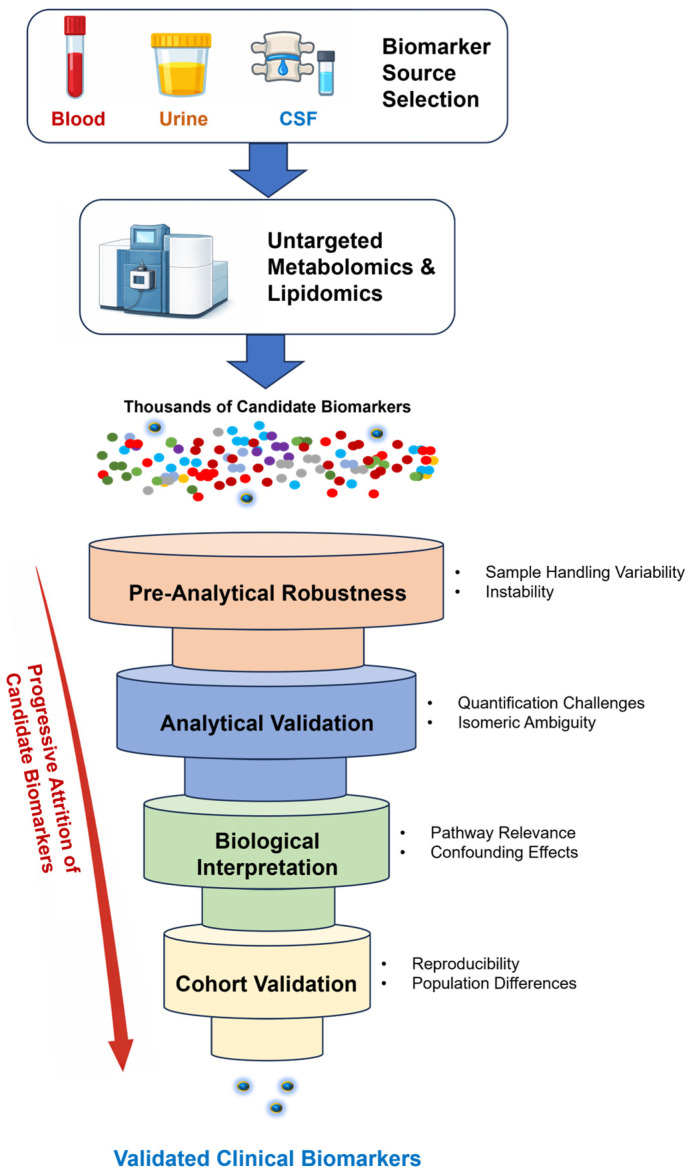
Systematic attrition of metabolic biomarkers across the translational pipeline. This schematic illustrates the progressive and non-random loss of metabolomics- and lipidomics-derived biomarker candidates as they advance from discovery to clinical application. Although large numbers of candidate features emerge during exploratory profiling, substantial attrition occurs at successive translational stages, including pre-analytical robustness, analytical validity, structural and annotation confidence, biological interpretability, and cohort validation. Attrition is shown to accelerate at predictable decision points where metabolite signals prove sensitive to handling conditions, dominated by ionization bias or semi-quantitative measurement, confounded by unresolved isomerism, or distorted by cohort-specific effects. The figure emphasizes that biomarker failure reflects structured translational constraints rather than biological irrelevance, underscoring the need for early go/no-go gating rather than post hoc statistical correction.

Metabolomics and lipidomics have reshaped biomedical research by enabling systematic, high-throughput measurement of small molecules across diverse biological systems. Over the past two decades, these approaches have generated thousands of candidate biomarkers for cancer, cardiometabolic disorders, inflammatory diseases, and neurodegeneration. Yet, despite this abundance, only a small fraction of metabolite-based biomarkers have achieved durable clinical implementation, and even fewer have demonstrated reproducible utility across independent cohorts and real-world clinical settings [1,2,3,4,5,6]. This pronounced discovery-to-implementation attrition is summarized in Figure 1, which situates metabolomics and lipidomics within the broader biomarker translational pipeline and highlights the stages at which candidate biomarkers are most frequently lost.

This persistent translational gap is often attributed to limited cohort size, insufficient statistical power, or the intrinsic biological complexity of metabolic regulation. However, accumulated evidence from biomarker research indicates that these explanations are incomplete. Across multiple disease areas, biomarkers frequently fail not because initial associations were incorrect, but because early studies did not adequately account for bias, pre-analytical variability, analytical uncertainty, and context-dependent performance that emerge during validation and clinical deployment [4,7,8,9,10]. These failures reflect structural weaknesses in study design and interpretation rather than isolated technical shortcomings.

A central misconception underlying metabolic biomarker research is the assumption that statistically significant concentration differences directly correspond to disease-specific biological signals. In practice, metabolite levels integrate a wide range of physiological and technical influences, including nutritional status, circadian rhythm, organ crosstalk, medication use, and systemic stress. Large-scale human metabolome studies have repeatedly demonstrated that such contextual factors introduce structured variability that can rival or exceed disease-associated effects [11,12]. Without explicit consideration of these factors, discovery-phase associations frequently collapse during external validation or clinical implementation [13,14,15,16,17]. Recurrent analytical and interpretative failure modes—spanning pre-analytical handling, analytical validity, and biological specificity—are consolidated into explicit, decision-oriented criteria in Table 1, which frames these vulnerabilities as early go/no-go considerations rather than post hoc explanations.

Methodological rigor and transparent reporting have therefore emerged as central determinants of successful translation. Reporting guidelines such as REMARK and STARD emphasize that inadequate study design, selective reporting, and unclear analytical workflows can substantially inflate perceived performance while obscuring fragility [18,19]. Parallel concerns have been raised across omics disciplines, where incomplete reporting and optimistic model development practices contribute to irreproducible findings and downstream validation failure [20]. In parallel, regulatory frameworks such as the FDA–NIH BEST Resource formalize the distinction between analytical validity, clinical validity, and clinical utility, underscoring that these requirements must be demonstrated sequentially rather than inferred from discovery data alone [21].

Metabolomics and lipidomics also face vulnerabilities that amplify these general translational challenges. Untargeted workflows are particularly sensitive to batch effects, ionization-dependent bias, and overfitting in high-dimensional data, all of which can generate optimistic but non-generalizable results if not explicitly addressed [22,23]. Even sophisticated multivariate or machine-learning models cannot compensate for unstable input features, and may instead encode cohort-specific artifacts that fail under external validation [24,25,26,27].

**Figure 2:**
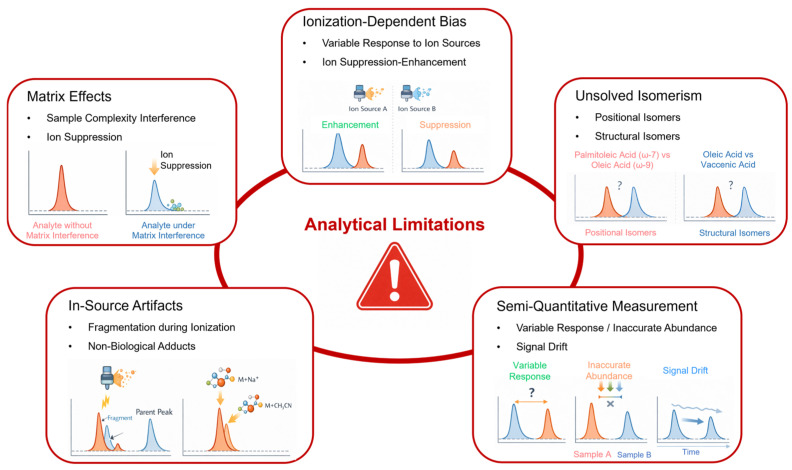
Analytical barriers undermining the clinical translation of metabolic biomarkers. This schematic illustrates major analytical vulnerabilities inherent to untargeted metabolomics and lipidomics workflows. Ionization-dependent bias and matrix effects are depicted as variable suppression or enhancement of analyte signals. Semi-quantitative measurement is illustrated by unstable signal responses and drift across analytical contexts. Unresolved positional and structural isomerism is exemplified using biologically relevant fatty acids, highlighting ambiguity in molecular assignment despite reproducible signals. In-source artifacts, including fragmentation during ionization and non-biological adduct formation, further distort apparent molecular identities. Collectively, these limitations generate analytically reproducible yet translationally fragile biomarker signals.

Lipidomics introduces further layers of complexity. Many lipid species exist as ensembles of structural and positional isomers that are indistinguishable by conventional mass spectrometry yet differ substantially in biological function. Misannotation, unresolved isomeric overlap, and in-source artifacts can profoundly distort downstream interpretation, particularly when single-lipid changes are promoted as mechanistic biomarkers [28,29,30]. Community-wide efforts to standardize lipid nomenclature and reporting further underscore how unresolved structural ambiguity remains a major barrier to reproducibility [31,32]. The analytical vulnerabilities that disproportionately undermine lipid biomarker translation—especially isomeric ambiguity, in-source artifacts, and ionization-dependent bias—are schematically summarized in Figure 2. Recent advances in ion-mobility and high-resolution workflows further underscore the extent to which conventional approaches underestimate lipid diversity, reinforcing the risk of overinterpretation in clinical contexts [33].

Importantly, biomarker attrition is not evenly distributed across the translational pipeline. While discovery studies routinely report strong associations, failures concentrate during analytical validation, external cohort testing, and assessments of clinical utility. This pattern mirrors long-standing observations across biomarker research, where promising candidates fail not because measurements were technically incorrect, but because their performance is highly context-dependent and incompatible with real-world clinical decision-making [5,14,34,35]. These stage-specific failure points are explicitly aligned with the pipeline overview in Figure 1 and the decision-gate framework summarized in Table 1.

This table summarizes recurrent failure modes that undermine the clinical translation of metabolomics- and lipidomics-based biomarkers and maps them to explicit decision gates across the translational pipeline. For each stage—pre-analytical handling, analytical validity, structural annotation, biological interpretation, and validation—the table highlights the dominant source of fragility, key diagnostic questions that should be addressed, and practical go/no-go implications. Rather than serving as a checklist for optimization, the framework is intended to function as an early filtering tool to deprioritize candidates that are incompatible with real-world clinical deployment. The table emphasizes that many biomarker failures are predictable consequences of unmet translational criteria rather than isolated technical shortcomings.

In this review, we argue that metabolomics and lipidomics have not failed as technologies but have been systematically overinterpreted as sources of standalone clinical biomarkers. Rather than treating metabolites as disease-specific indicators, successful clinical translation requires reframing metabolic measurements as context-sensitive reporters whose utility depends on analytical robustness, biological interpretability, and alignment with clinical decision-making. Building on established biomarker evaluation frameworks, we present a practical roadmap that dissects recurrent failure modes across pre-analytical handling, analytical quantification, structural annotation, statistical inference, and cohort validation, and translates them into explicit decision gates for clinical advancement (Table 1), mapped onto the broader translational pipeline (Figure 1).

## 2. Defining Clinical Translation for Metabolic Biomarkers

### 2.1. A Biomarker Is Not a Clinical Decision Tool

In metabolomics and lipidomics research, the term biomarker is frequently used with limited precision, often referring to any molecular feature that differs statistically between cases and controls. However, from a clinical and regulatory perspective, a biomarker is not defined by statistical association alone, but by its ability to inform or alter a clinical decision in a reproducible, interpretable, and context-appropriate manner [1,3,36,37]. This distinction is fundamental, as many metabolite candidates satisfy discovery-phase criteria yet fail to meet the requirements for clinical deployment.

In clinical practice, biomarkers are never interpreted in isolation. Instead, they are embedded within diagnostic, prognostic, or therapeutic decision frameworks that require clearly defined use cases, decision thresholds, and predictable performance across patient populations. A metabolite that demonstrates a statistically significant difference between disease and control groups may still lack clinical value if it does not improve diagnostic accuracy beyond existing standards, fails to stratify risk in a clinically actionable manner, or cannot be interpreted consistently across laboratories and clinical settings [1,3,13,26,27].

From a regulatory standpoint, this distinction becomes even more stringent. Regulatory agencies evaluate biomarkers not as biological signals but as components of medical tests, requiring independent evidence of analytical validity, clinical validity, and clinical utility. This framework aligns directly with regulatory principles outlined in the FDA–NIH BEST Resource and the European IVDR framework, which require sequential demonstration of analytical validity, clinical validity, and clinical utility. The translational gates proposed in this review explicitly mirror these regulatory stages. Within this framework, uncertainty in metabolite identity, instability during sample handling, or context-dependent variability is not a minor technical limitation but a fundamental barrier to approval and adoption [6,21].

### 2.2. Re-Examining the Phases of Biomarker Translation

The classical biomarker development pipeline—comprising discovery, analytical validation, clinical validation, and assessment of clinical utility—has been well described for over two decades [1,3] and is summarized in Figure 1. Despite this established framework, metabolomics and lipidomics studies frequently compress or conflate these phases, implicitly assuming that strong discovery-phase associations will naturally translate into downstream success. Evidence from both general biomarker research and metabolomics-specific investigations indicates that this assumption is rarely justified [4,9,10].

During the discovery phase, untargeted metabolomics and lipidomics excel at identifying large numbers of candidate features. However, these features are often reported with incomplete structural annotation, semi-quantitative measurements, and limited consideration of pre-analytical or analytical variability [38,39,40]. While such conditions are acceptable for hypothesis generation, they are fundamentally incompatible with the expectations of analytical validation.

Analytical validation represents the first major attrition point for metabolic biomarkers. At this stage, issues such as ionization bias, matrix effects, batch drift, and dependence on platform-specific workflows become apparent [22,41,42]. Metabolite signals that appear stable in controlled discovery settings often exhibit unacceptable variability when measured across different instruments, laboratories, or sample matrices.

Clinical validation introduces additional complexity. Metabolite concentrations are shaped by age, sex, diet, circadian rhythm, comorbidities, and medication use, all of which vary substantially between cohorts and clinical environments [11,12,16,17]. Consequently, cut-off values derived in one cohort frequently fail in external populations, leading to marked losses in sensitivity or specificity [10,14].

Finally, clinical utility—the ability of a biomarker to improve patient outcomes or meaningfully guide clinical decisions—is seldom addressed explicitly in metabolomics studies. Without this framing, even analytically and clinically valid biomarkers may remain clinically irrelevant [34,35].

## 3. Pre-Analytical Robustness: Before You Measure Anything

Pre-analytical variability represents the earliest and often most consequential source of failure in metabolomics- and lipidomics-based biomarker development. From a clinical perspective, pre-analytical factors encompass all processes occurring before analytical measurement, including patient preparation, sample collection, handling, storage, and transport. Although often treated as secondary technical details in discovery studies, these variables exert systematic and frequently irreversible effects on metabolite composition and concentration, directly undermining downstream analytical validity and clinical interpretability [7,8,38,39]. Accordingly, pre-analytical robustness constitutes the first translational decision gate formalized in Table 1.

Crucially, pre-analytical robustness is not a matter of optimization but of eligibility for clinical translation. A metabolite whose measured concentration is highly sensitive to routine clinical handling conditions cannot realistically function as a decision-grade biomarker, regardless of its apparent biological relevance under controlled research settings. From a translational standpoint, pre-analytical sensitivity therefore represents a hard constraint rather than a parameter to be adjusted post hoc through statistical correction. Treating pre-analytical instability as a downstream nuisance risks advancing intrinsically fragile biomarkers into stages of development where failure becomes inevitable and costly [9,10].

### 3.1. Biological Context and Sample Origin

The biological meaning of a metabolite signal is inseparable from its compartment of origin. In clinical metabolomics, plasma and serum are the most frequently analyzed matrices owing to their accessibility and compatibility with routine workflows. However, circulating metabolites reflect an integrated systemic milieu shaped by hepatic metabolism, renal clearance, inter-organ exchange, endocrine regulation, and immune activation, rather than localized disease-specific processes in isolation [11,12,17,43].

This limitation is particularly relevant for diseases in which tissue-specific metabolic alterations are hypothesized to drive pathogenesis, such as cancer or neurodegenerative disorders. In such contexts, plasma-based metabolite changes often represent downstream or compensatory responses—including inflammation, cachexia, oxidative stress, or treatment-related effects—rather than direct readouts of disease-specific metabolic pathways. Without explicit consideration of this biological context, metabolite associations risk being misinterpreted as disease-specific biomarkers when they instead report generalized physiological perturbation [16,43,44].

Therefore, clinical translation requires that the relationship between the sampled biological compartment and the intended clinical use be defined explicitly. Biomarkers intended for diagnosis, risk stratification, or treatment monitoring must be interpretable within the biological constraints of the sampled matrix. Failure to align biological context with clinical intent is a common but underappreciated cause of translational collapse and is therefore captured as an explicit eligibility consideration in Table 1.

### 3.2. Patient Preparation and Physiological State

Unlike genomic markers, metabolite concentrations are acutely sensitive to physiological state. Fasting status, recent dietary intake, circadian rhythm, physical activity, and acute psychological or inflammatory stress can induce metabolic changes that rival or exceed those associated with disease. Large-scale population metabolomics studies have consistently demonstrated that such factors introduce structured, non-random variability into metabolic profiles [11,12,16,45].

From a clinical perspective, this sensitivity raises a fundamental feasibility question: can the biomarker be measured reliably under real-world conditions? If a metabolite requires strict fasting, narrow sampling windows, or exclusion of commonly prescribed medications to maintain interpretability, its clinical utility is inherently limited. Regulatory evaluation implicitly incorporates these considerations, favoring biomarkers whose performance remains robust across routine clinical variability rather than optimized experimental conditions [3,15].

Importantly, post hoc statistical adjustment cannot fully compensate for poorly controlled physiological variability. If biomarker interpretability depends on retrospective correction for diet, timing, or stress, its deployment in routine clinical workflows becomes impractical. Consequently, physiological robustness should be evaluated early and treated as a translational gate rather than a secondary analytical refinement.

### 3.3. Sample Collection and Handling

Sample collection procedures introduce additional layers of pre-analytical variability. The choice of anticoagulant, clotting time for serum, temperature during processing, and delays between collection and stabilization all influence metabolite stability and apparent abundance. Lipid species are particularly vulnerable to enzymatic remodeling, oxidation, and hydrolysis during even brief deviations from standardized handling protocols [28,38,46,47]. Comprehensive workflow analyses in lipidomics have demonstrated that variability introduced during sample preparation, extraction, and handling can dominate downstream analytical outcomes, reinforcing pre-analytical robustness as a critical determinant of translational viability [48].

Freeze–thaw cycles further complicate interpretation. Although often dismissed as minor technical nuisances, repeated freeze–thaw events can selectively degrade or transform metabolites and lipids, generating artifactual differences that mimic biological effects. Critically, these effects are not uniform across metabolite classes, introducing systematic bias rather than random error. In a clinical environment—where samples may undergo variable handling before analysis—such sensitivities represent a major obstacle to reproducibility and cross-site comparability [49].

### 3.4. Storage, Stability, and Longitudinal Comparability

Clinical translation frequently requires longitudinal sample analysis, whether for validation across cohorts or for monitoring disease progression and treatment response. Under these conditions, storage stability becomes a defining criterion for biomarker viability. Metabolites that degrade, oxidize, or interconvert during storage—even under ostensibly appropriate conditions—cannot support reliable longitudinal comparisons [38,39].

For lipidomics, storage-related artifacts are especially problematic. Oxidative processes can generate secondary lipid species that are difficult to distinguish from endogenous signaling molecules, particularly when high-resolution mass spectrometry is applied without orthogonal structural confirmation [28,50,51]. Without explicit stability assessment, such artifacts may be incorporated into biomarker models and falsely attributed to disease-related biological processes.

Long-term biobanked samples introduce additional uncertainty. Variability in storage duration, temperature fluctuations, and historical handling practices can introduce cohort-dependent bias that is difficult to detect retrospectively. Biomarkers intended for longitudinal or multicenter use must therefore demonstrate stability under realistic storage conditions representative of clinical practice.

### 3.5. Pre-Analytical Variability as a Go/No-Go Criterion

From a translational and regulatory perspective, pre-analytical sensitivity should be treated as a go/no-go criterion rather than a downstream nuisance to be statistically corrected. If a metabolite’s signal cannot withstand realistic clinical handling conditions, it should be deprioritized early in development. Attempts to salvage such candidates through normalization, batch correction, or increasingly complex modeling often shift—rather than resolve—the underlying problem [10,35].

Therefore, a practical roadmap for clinical translation requires systematic documentation and stress-testing of pre-analytical robustness. This includes explicit reporting of patient preparation, collection protocols, handling timelines, storage conditions, and stability assessments. Such transparency not only facilitates reproducibility but also enables informed evaluation by clinicians, laboratory directors, and regulatory bodies tasked with determining whether a biomarker is fit for clinical use. The corresponding eligibility criteria are summarized in Table 1, reinforcing pre-analytical robustness as the first and most decisive translational gate.

## 4. Analytical Validity: Quantification Is Not What You Think

Analytical validity is often treated as a technical checkpoint to be cleared after biomarker discovery. However, in metabolomics and lipidomics, analytical considerations fundamentally shape the meaning of the measured signal itself. Unlike classical clinical chemistry assays, where analyte identity and quantification are tightly defined, most metabolomics workflows operate under semi-quantitative conditions that are frequently misinterpreted as absolute or decision-grade measurements [39,41,50,52]. For this reason, analytical validity is framed as a distinct translational gate in Table 1, rather than a downstream technical refinement.

From a translational perspective, the central challenge is not a lack of analytical sophistication but a mismatch between what mass spectrometry-based measurements can reliably deliver and what clinical decision-making requires. This mismatch becomes particularly evident when metabolite concentrations are implicitly treated as stable, comparable quantities across samples, batches, platforms, and laboratories.

### 4.1. Ionization Bias and Matrix Effects

Electrospray ionization (ESI), the dominant ionization method in metabolomics and lipidomics, is inherently selective. Ionization efficiency varies widely across metabolite classes, lipid headgroups, acyl-chain composition, and even subtle structural features such as double-bond position or oxidation state. Consequently, signal intensity is not a direct proxy for molar abundance, even within a single analytical run [47,48,50,51].

Matrix effects further compound this problem. Co-eluting compounds, salts, and endogenous matrix components can suppress or enhance ionization in a sample-dependent manner, producing apparent differences that reflect analytical context rather than underlying biology. Importantly, these effects are structured rather than random: specific lipid classes or metabolite families are consistently favored or penalized under particular chromatographic and ionization conditions [38,41,53].

In tightly controlled discovery studies, such biases may be tolerable when the goal is relative comparison within a single batch. However, in clinical translation, where samples originate from heterogeneous settings and are processed over extended periods, ionization bias undermines the assumption that measured changes reflect reproducible biological differences. The interaction between ionization bias and matrix effects—and how these phenomena can generate apparent but fragile biomarker signals—is illustrated schematically in Figure 2.

### 4.2. The Limits of Internal Standards

The use of internal standards is widely promoted as a solution to analytical variability. While internal standards are essential for quality control and normalization, their corrective power is often overstated. In practice, most metabolomics and lipidomics studies rely on class-matched or surrogate standards rather than isotopically labeled analogs for each analyte of interest. Even when isotopic standards are available, they may not adequately account for structural heterogeneity, unresolved isomerism, or differential matrix effects [42,47,54].

In lipidomics, these limitations are particularly pronounced. A single phosphatidylcholine internal standard cannot faithfully correct for the ionization behavior of a diverse ensemble of phosphatidylcholine species differing in chain length, degree of unsaturation, or oxidative modification. As a result, normalization may reduce gross variability while preserving systematic bias within and across lipid subclasses [47,51,55].

From a regulatory standpoint, these limitations are critical. Analytical validation requires evidence that an assay measures what it claims to measure with defined accuracy and precision. Semi-quantitative normalization strategies that obscure analyte-specific variability rather than resolving it are unlikely to meet this standard [21,40].

### 4.3. Batch Effects and the Illusion of Correction

Batch effects are ubiquitous in large-scale metabolomics studies and are commonly addressed using quality control (QC)-based normalization strategies. While QC samples are invaluable for monitoring instrument performance and detecting drift, they cannot fully compensate for structural differences between batches arising from changes in sample composition, matrix effects, or analytical conditions [39,56].

A critical but underappreciated issue is that batch correction methods can inadvertently reinforce false biological patterns. When disease and control samples are unevenly distributed across batches, normalization algorithms may amplify group differences that are, in reality, artifacts of analytical structure. In such cases, statistical significance reflects successful variance correction rather than faithful measurement of biology [10,22,57].

For clinical translation, this poses a serious risk. A biomarker whose apparent performance depends on batch-specific correction is unlikely to reproduce when measured prospectively or across independent laboratories, where batch composition and analytical context necessarily differ.

### 4.4. Semi-Quantitative Data in a Quantitative World

Clinical decision-making relies on defined thresholds, reference ranges, and reproducible metrics. By contrast, most metabolomics datasets provide relative intensities or normalized values that lack intrinsic meaning outside the context of the study in which they were generated. Treating such data as interchangeable with absolute concentrations introduces a semantic error with tangible clinical consequences.

This disconnect becomes evident when metabolomics-derived cut-offs are proposed for diagnosis or prognosis without acknowledging their dependence on platform, protocol, and analytical context. Even modest changes in instrumentation, chromatography, or data processing can shift relative intensities sufficiently to invalidate fixed thresholds [27,41,42,58].

A translational roadmap must therefore distinguish explicitly between exploratory measurement and decision-grade quantification. Metabolites that cannot be quantified with acceptable accuracy and robustness under clinically realistic conditions should not be advanced as standalone biomarkers.

### 4.5. Analytical Validity as a Translational Filter

Rather than attempting to retrofit analytical rigor onto discovery-phase findings, analytical validity should function as an early translational filter. Metabolites whose measurement is dominated by ionization bias, matrix dependence, or batch-specific behavior are poor candidates for clinical deployment, regardless of their statistical association with disease.

From a regulatory perspective, transparency is essential. Reporting should explicitly state the degree of quantification confidence, the nature and limitations of internal standardization, and the expected variability across platforms and laboratories. Such disclosure enables clinicians and regulators to judge whether a reported biomarker signal is compatible with the demands of clinical testing [21,40,52].

In summary, the analytical challenges of metabolomics and lipidomics do not represent minor technical hurdles; they define the boundaries of what can be translated into clinical practice. Recognizing and respecting these boundaries is a prerequisite for meaningful progress.

## 5. Structural and Annotation Confidence: Knowing What You Are Measuring

Structural and annotation confidence is a prerequisite for any biomarker intended for clinical use. However, in metabolomics and lipidomics, molecular identity is frequently inferred rather than established, and this uncertainty is often obscured by high-resolution mass spectrometry readouts that convey a misleading sense of precision. From a translational standpoint, ambiguity in analyte identity is not a minor technical inconvenience but a fundamental liability that undermines analytical validity, biological interpretation, and regulatory acceptability [40,42,51]. For this reason, minimum requirements for structural definition and annotation confidence are treated as a dedicated translational gate in Table 1.

Clinical assays are expected to measure a clearly defined molecular entity. When the identity of that entity is uncertain or context-dependent, downstream claims regarding disease association, mechanism, or clinical utility become difficult—if not impossible—to substantiate. In metabolomics-based biomarker research, this gap between nominal identification and molecular certainty represents a recurrent but underacknowledged source of translational failure.

### 5.1. Levels of Annotation and Overconfidence in Identification

Most metabolomics and lipidomics studies rely on putative annotation based on accurate mass, retention time, and tandem mass spectra matched against reference databases. While these approaches enable high-throughput discovery, they do not guarantee unambiguous identification. Multiple candidate structures may share indistinguishable spectral features, particularly when isomeric or isobaric species are involved [42,51,59,60,61]. Tools for retention-time prediction and database-driven annotation can improve consistency but do not eliminate fundamental ambiguity when chemical space is dense or reference standards are absent [62]. The importance of retention time as an orthogonal identifier has been demonstrated through curated serum lipid libraries combining accurate mass and chromatographic behavior, underscoring how annotation confidence depends on more than mass accuracy alone [63].

Despite this limitation, putative annotations are frequently reported using definitive nomenclature, implicitly conveying a level of certainty that is not supported by the underlying data. This practice becomes especially problematic when such features are advanced as biomarkers, as clinical and regulatory frameworks require an explicit definition of what is being measured. A biomarker whose identity depends primarily on database matching rather than confirmed structural characterization cannot be assumed to behave consistently across analytical platforms or laboratories.

From a translational perspective, failure to distinguish clearly between putative and confirmed identification propagates uncertainty downstream, contaminating analytical validation, biological interpretation, and external validation efforts.

### 5.2. Isomerism and the Collapse of Biological Meaning

Lipidomics is particularly vulnerable to structural ambiguity due to the prevalence of isomeric species. Lipids sharing the same nominal mass and elemental composition may differ in sn-position, double-bond location, cis/trans configuration, or oxidative modification—differences that can profoundly influence membrane properties, signaling interactions, and metabolic fate [29,47,64,65].

Conventional LC–MS workflows often fail to resolve these distinctions, leading to aggregation of multiple molecular entities under a single reported feature. When such aggregated signals are interpreted as discrete biological markers, the resulting conclusions conflate heterogeneous molecular behaviors. This collapse of structural resolution into simplified readouts undermines mechanistic inference and introduces substantial uncertainty into any proposed clinical application.

The analytical consequences of unresolved isomerism—particularly for lipid biomarkers—are schematically summarized in Figure 2, which illustrates how distinct molecular species can generate overlapping signals yet diverge in biological function.

From a regulatory perspective, this issue is decisive. An assay that measures a variable mixture of molecular species under a single name lacks the analytical specificity required for clinical approval. Without explicit acknowledgment and control of isomeric complexity, lipid-based biomarkers remain intrinsically unstable. Community standards for nomenclature and reporting reflect this requirement for explicit structural definition [31,32].

### 5.3. Artifacts and In-Source Transformation

Beyond annotation uncertainty, mass spectrometry-based workflows are susceptible to the generation of analytical artifacts. In-source fragmentation, adduct formation, and oxidation can produce signals that mimic endogenous metabolites or lipids, particularly under high-energy ionization conditions. These artifacts may vary systematically with instrument settings, sample composition, or analytical batch, creating reproducible but biologically meaningless patterns [47,49,50].

In lipidomics, oxidative artifacts are of particular concern. Lipid oxidation can occur during sample handling, storage, or ionization, generating species that resemble bona fide signaling molecules such as oxylipins. Without rigorous controls and orthogonal confirmation, these artifactual signals may be misclassified as disease-associated metabolites, propagating false biological narratives into biomarker development pipelines [28,49].

Crucially, the reproducibility of such artifacts does not confer biological validity. From a translational standpoint, consistent measurement of the wrong entity is not a success but a failure of analytical specificity.

### 5.4. Annotation Confidence as a Regulatory Requirement

Regulatory evaluation of in vitro diagnostics emphasizes analytical specificity, traceability, and reproducibility. These criteria presuppose that the analyte of interest is clearly defined and consistently measurable. In this context, ambiguous annotation is not a minor reporting deficiency but a substantive barrier to approval.

Therefore, a translational roadmap demands explicit reporting of annotation confidence, including the level of structural confirmation achieved and the potential presence of unresolved isomers or in-source artifacts. Where definitive identification is not feasible, limitations should be stated transparently, and claims regarding biological mechanism or clinical applicability should be correspondingly constrained. These expectations align with emerging community standards in metabolomics and lipidomics [31,32,40].

### 5.5. Implications for Biomarker Development

Structural and annotation uncertainty explains, in part, why many metabolite-based biomarkers perform well in discovery cohorts yet fail during external validation. Apparent associations may reflect cohort-specific mixtures of molecular species or analytical artifacts rather than stable, disease-linked entities. When measured under different conditions or with alternative workflows, the same nominal feature may represent a different molecular composition, leading to inconsistent results.

Within a decision-gate framework, annotation confidence should therefore function as an early filter. Metabolites or lipids whose identity cannot be defined with sufficient clarity should be deprioritized as standalone biomarkers. Alternatively, they may be incorporated into multi-metabolite patterns or pathway-level summaries, where individual ambiguity is partially mitigated by collective behavior. The corresponding translational criteria are summarized in Table 1, reinforcing molecular definition as a non-negotiable requirement for clinical advancement.

## 6. Statistical Association vs. Biological Meaning: When Significance Is Not Mechanism

Statistical association is the dominant currency of biomarker discovery. In metabolomics and lipidomics, advances in high-dimensional data acquisition and machine-learning approaches have further amplified the ability to identify features that distinguish cases from controls with impressive statistical metrics. Yet, a strong association does not guarantee biological relevance, nor does it ensure clinical durability. Repeated validation failure highlights a fundamental disconnect between statistical significance and biological meaning [10,20].

### 6.1. Multiple Testing, Overfitting, and Optimism Bias

Untargeted metabolomics inherently involves testing hundreds to thousands of features simultaneously. Even with appropriate multiple-testing correction, the risk of overfitting remains substantial, particularly when feature selection, model tuning, and performance evaluation are conducted within the same dataset. Cross-validation does not fully mitigate this risk when cohort sizes are modest or confounding variables are unevenly distributed [20,23]. Selective reporting further exacerbates optimism bias, and optimistic internal validation can inflate apparent discrimination even when generalization is poor [26,66]. From a translational standpoint, reported performance metrics often reflect best-case scenarios rather than realistic clinical operation.

### 6.2. Disease-Specificity Collapse Under Systemic Stress

A central assumption in biomarker research is that disease-associated changes are distinguishable from background physiological variation. In metabolomics, this assumption is frequently violated. Many metabolites respond similarly to diverse pathological and non-pathological stressors, including inflammation, hypoxia, cachexia, infection, and aging [16,17,43]. This disease-specificity collapse is particularly evident in plasma-based studies, where circulating metabolites integrate signals from multiple organs and regulatory systems. Patterns are often dominated by systemic responses rather than localized disease mechanisms, rendering single-metabolite markers inherently unstable across clinical contexts.

### 6.3. Concentration Versus Flux: The Limits of Static Measurement

Most metabolomics studies rely on static concentration measurements obtained from single time points. Such snapshots provide limited insight into dynamic metabolic processes. Changes in concentration may reflect altered production, consumption, transport, or compartmentalization, none of which can be inferred reliably from concentration alone [16,43]. Acylcarnitines exemplify this limitation. Often interpreted as markers of discrete enzymatic defects, they more accurately report flux imbalance or metabolic overflow under stress. Misinterpreting such signals as disease-specific concentration markers leads to fragile mechanistic claims and unstable biomarkers. This limitation has been emphasized in pharmacometabonomics, where metabolite profiles are interpreted as indicators of dynamic metabolic state and individual response capacity rather than fixed concentration markers, highlighting the gap between static measurement and underlying biological flux [67]. Stable isotope tracing and kinetic modeling approaches provide a more direct assessment of metabolic flux and may overcome limitations inherent to static concentration measurements. However, such approaches introduce additional analytical complexity and standardization challenges that must be reconciled before routine clinical deployment.

### 6.4. Model Performance Does Not Equal Clinical Meaning

Multivariate models and machine-learning approaches can achieve high discriminatory performance in discovery cohorts. However, such performance does not imply robust biological insight or clinical readiness. Models may exploit batch structure, cohort-specific correlations, or surrogate signals unrelated to the disease mechanism [20,27]. Moreover, conventional metrics can obscure whether a model meaningfully changes clinical decisions: improvements in area under the curve (AUC) may be small despite statistically significant associations, while clinically relevant impact may be better captured by reclassification metrics and decision-analytic evaluation [24,68,69]. Therefore, regulatory and clinical frameworks emphasize interpretability, reproducibility, and external validation, and increasingly require transparent reporting of model development and evaluation [27,66].

### 6.5. Implications for Clinical Translation

Statistical association should be viewed as an entry point rather than an endpoint in biomarker development. Translational success requires progression beyond significance toward biological plausibility, specificity, and mechanistic coherence. This transition demands explicit consideration of metabolic context, confounders, and validation strategies aligned with real-world clinical complexity. The disconnect between statistical association and biological relevance has been widely recognized in omics-based biomarker research, where reliance on algorithmic significance without expert contextual interpretation contributes to poor reproducibility and translational failure [70]. The corresponding decision criteria are summarized in Table 1.

## 7. From Discovery to Validation: Why Cohorts Kill Biomarkers

The transition from discovery to validation represents the most severe attrition point in metabolic biomarker development. While discovery studies frequently report strong discriminatory performance, these findings often deteriorate or disappear entirely when tested in independent cohorts. This pattern is not anomalous but systematic, reflecting the sensitivity of metabolite signals to cohort-specific factors that are difficult to control or fully characterize [1,3,10]. From a translational perspective, validation failure is rarely surprising. Instead, it exposes assumptions made during discovery that do not hold under real-world clinical conditions.

### 7.1. Cohort Shift and Hidden Confounders

Cohort shift refers to systematic differences between discovery and validation populations that alter biomarker performance. In metabolomics and lipidomics, such shifts arise from variations in demographics, clinical practice, comorbidities, medication use, diet, and sample handling protocols. Even when cohorts share nominal diagnostic categories, their metabolic landscapes may differ substantially due to these contextual factors [11,12,16,22]. Hidden confounders are particularly problematic. Differences in treatment regimens, disease stage distribution, or supportive care practices can introduce metabolic changes unrelated to the biological process of interest. When such factors are unevenly distributed between cohorts, apparent biomarker performance may reflect cohort composition rather than disease biology [7,8].

### 7.2. External Validation: Necessary but Not Sufficient

External validation is widely recognized as a critical step in biomarker development. However, the mere presence of an external cohort does not guarantee meaningful validation. When validation datasets closely mirror discovery cohorts in sample handling, analytical platform, and population characteristics, they may fail to reveal underlying fragility [1,3]. Conversely, when validation cohorts differ substantially—as is common in multicenter or prospective studies—performance metrics often degrade sharply. Sensitivity and specificity may shift, optimal cut-off values may change, and previously significant features may lose discriminatory power altogether. From a regulatory standpoint, such instability is unacceptable for clinical deployment. Accordingly, synthesis approaches that evaluate performance across multiple external validations and quantify heterogeneity are increasingly important for judging durability [71].

### 7.3. The Cut-Off Problem

Clinical implementation requires defined thresholds that trigger action. In metabolomics studies, cut-offs are often derived empirically from discovery cohorts using statistical optimization. However, these thresholds are inherently cohort-specific, reflecting the distribution of metabolite values under particular analytical and clinical conditions [1,10]. When applied to new populations, cut-offs frequently lose validity. Adjusting thresholds post hoc to restore performance may salvage statistical metrics but undermines clinical interpretability and regulatory credibility. A biomarker that requires continuous recalibration across cohorts is incompatible with routine clinical use, particularly when the model’s incremental value is evaluated using reclassification or net benefit frameworks [24,68].

### 7.4. Prospective Studies and Real-World Performance

Retrospective studies dominate the metabolomics literature, yet prospective validation is essential for assessing real-world performance. Prospective studies introduce variability absent from retrospective analyses, including variation in sample handling, patient behavior, and clinical workflows [3]. Biomarkers that perform well retrospectively may underperform prospectively, revealing dependencies on tightly controlled conditions that are unrealistic in routine practice. From a clinical and regulatory perspective, prospective evidence carries disproportionate weight as it most closely reflects intended use.

### 7.5. Validation as a Stress Test, Not a Formality

Within a guide-type framework, validation should be viewed as a stress test rather than a confirmatory formality. The goal is not to reproduce discovery performance but to identify failure modes before clinical deployment. Robust biomarkers are those that remain interpretable and reliable under adversity. These criteria are consolidated in Table 1 and aligned with the translational pipeline in Figure 1.

## 8. What Actually Works: Moving Beyond Single-Metabolite Thinking

The cumulative evidence reviewed above supports a clear conclusion: single-metabolite biomarkers are structurally ill-suited for reliable clinical translation in most contexts. This limitation reflects not a failure of metabolomics or lipidomics as disciplines, but a mismatch between the complexity of metabolic regulation and the simplicity implied by single-analyte decision rules [16,44].

### 8.1. Multi-Metabolite Panels and Pattern-Based Signatures

Multi-metabolite panels mitigate vulnerabilities of individual analytes by integrating complementary signals. Properly constructed panels reduce sensitivity to pre-analytical variability, analytical bias, and biological noise. Redundancy within panels enhances robustness by buffering instability in individual components. Pattern-based signatures—defined by relative relationships rather than absolute concentrations—further increase resilience by exploiting internal normalization within metabolic networks. Panels that maintain performance across cohorts and platforms are more clinically valuable than single markers with large discovery-phase effect sizes but poor reproducibility. Importantly, multi-metabolite panels may propagate analytical variability when individual components exhibit independent measurement error. Error accumulation can amplify variance in composite scores if analytes are not analytically harmonized. Therefore, panel construction should incorporate feature stability analysis, variance decomposition, and assessment of error propagation across batches and platforms. Examples of successful translation illustrate these principles. Newborn screening programs using targeted acylcarnitine panels for inborn errors of metabolism represent one of the most durable implementations of metabolomics-based diagnostics. Similarly, phenylalanine quantification for phenylketonuria (PKU) screening demonstrates how strict analytical validation, molecular specificity, and clear clinical actionability enable long-term clinical adoption. Targeted lipid panels in cardiovascular risk stratification further exemplify metabolite-based measurements that align analytical rigor with decision-making frameworks.

### 8.2. Model-Based and AI-Assisted Approaches: Promise and Pitfalls

Machine-learning methods can integrate weak individual signals into robust composite predictors. However, modeling does not circumvent foundational requirements of analytical validity and biological interpretability. Models trained on unstable or artifact-prone features inevitably encode these weaknesses, regardless of algorithmic sophistication [20]. Performance metrics derived from internal cross-validation frequently overestimate real-world utility, particularly in the presence of cohort-specific confounders. Techniques such as nested cross-validation and stability selection are particularly important to prevent information leakage and optimistic bias during feature selection and model tuning. Regulatory frameworks increasingly emphasize transparency, interpretability, and external validation for AI-assisted clinical tools, including clearer reporting expectations [27,66].

### 8.3. Mechanism-Informed Biomarkers and Flux-Oriented Interpretation

Anchoring biomarker development in mechanistic understanding provides an alternative to isolated concentration-based markers. Interpreting metabolites as reporters of pathway activity or metabolic flux aligns more closely with biological reality [16,43]. Such mechanism-informed approaches improve interpretability for clinicians and strengthen biological plausibility for regulators, even when mechanistic detail remains incomplete.

### 8.4. Reporting Standards and Reproducibility as Enablers

Translation of multi-metabolite and model-based biomarkers depends critically on rigorous reporting standards. Transparent disclosure of pre-analytical handling, analytical workflows, annotation confidence, and model development enables meaningful evaluation and replication. Emerging reporting frameworks for prediction models represent a critical step toward aligning research practice with clinical and regulatory expectations [27,66,71].

### 8.5. From Strategy to Practice

Moving beyond single-metabolite thinking does not guarantee success, but it addresses many structural weaknesses underlying biomarker failure. Multi-metabolite panels, pattern-based signatures, and mechanism-informed models offer more realistic paths to clinical utility when embedded within a disciplined translational framework.

## 9. A Practical Roadmap for Clinical Translation: From Discovery toDecision-Grade Biomarkers

The preceding sections identify recurrent failure modes that undermine the clinical translation of metabolomics- and lipidomics-based biomarkers. Importantly, these failures cluster at predictable stages of the translational pipeline. This section consolidates these insights into a practical roadmap intended to guide investigators, clinicians, laboratory directors, and regulators in evaluating whether a metabolic biomarker candidate is suitable for advancement. A consolidated checklist of these translational decision points is provided in Table 1, aligned with the broader pipeline context summarized in Figure 1.

### 9.1. Pre-Analytical Eligibility

Before analytical optimization or statistical modeling, candidate biomarkers must be evaluated for pre-analytical eligibility under conditions approximating routine clinical practice. This requires explicit alignment between the sampled biological compartment and the intended clinical use, as well as evidence that measured levels remain interpretable across realistic variation in fasting status, circadian timing, and routine handling. Failure at this stage represents a structural barrier to translation rather than a technical limitation [38,39]. As indicative benchmarks (not universal rules), coefficients of variation exceeding ~20% across batches, or pre-analytical shifts exceeding two-fold under routine handling conditions, may indicate incompatibility with decision-grade clinical testing. Similarly, biomarkers lacking at least MSI level 2 structural confidence should not be advanced as standalone clinical analytes. Importantly, each gate in this roadmap corresponds conceptually to regulatory evaluation domains defined by the FDA–NIH BEST framework and the European IVDR, namely analytical validity, clinical validity, and clinical utility. The alignment presented here is intended to facilitate early translational design that anticipates regulatory expectations rather than retrofitting compliance at later stages.

Gate criterion: Advance only analytes that retain biological interpretability under clinically realistic collection, processing, and storage variability.

### 9.2. Analytical Validity Gate

Analytical evaluation must distinguish clearly between exploratory measurement and decision-grade quantification. Given the pervasive influence of ionization bias, matrix effects, and platform dependence, analytical validity requires demonstrating stability across batches, platforms, and laboratories at a level compatible with clinical decision-making [40,41,42,50]. As an indicative analytical benchmark, drift exceeding predefined quality-control limits (e.g., >15–20% deviation in QC samples across analytical runs) may indicate inadequate stability for decision-grade implementation.

Gate criterion: Advance only candidates whose analytical performance is sufficiently robust for the intended clinical application.

### 9.3. Structural and Annotation Confidence Requirement

Clinical biomarkers must correspond to clearly defined molecular entities. Ambiguity arising from unresolved isomers, adducts, or artifacts undermines analytical specificity and biological interpretation [31,32,47,51].

Gate criterion: Advance only biomarkers with sufficient molecular definition to ensure analytical specificity and reproducibility.

### 9.4. Biological Interpretability and Specificity Assessment

Biomarkers should demonstrate plausible linkage to disease-relevant biology and retain specificity when evaluated against common confounders and differential diagnoses [16,17,43].

Gate criterion: Advance candidates only when the biological interpretation is coherent and compatible with the clinical question.

### 9.5. Validation Strategy and Clinical Utility Alignment

Validation must be designed as a stress test rather than a confirmatory exercise. Robust biomarkers maintain interpretable performance across heterogeneous cohorts and real-world clinical workflows and provide clear incremental clinical utility beyond existing standards [1,3]. Where possible, clinical value should be judged using evaluation frameworks that explicitly connect predictive performance to decision-making consequences, rather than discrimination alone [24,66,68].

Gate criterion: Advance only biomarkers that demonstrate stable performance and meaningful clinical impact.

## 10. Conclusions and Outlook

Metabolomics and lipidomics have transformed our capacity to interrogate metabolic states across health and disease, offering unprecedented breadth and sensitivity in molecular measurement. Yet, the persistent failure of metabolite-based biomarkers to achieve durable clinical translation highlights a critical mismatch between discovery-driven research practices and the realities of clinical and regulatory deployment. This review argues that this gap does not reflect a fundamental inadequacy of metabolomics or lipidomics as technologies, but rather systematic overinterpretation of their outputs as standalone clinical biomarkers.

Across the translational pipeline, recurrent failure modes emerge with striking regularity. Pre-analytical variability undermines interpretability before measurement even begins; analytical semi-quantitation and ionization bias distort apparent abundance; structural ambiguity collapses molecular specificity; statistical association is mistaken for biological mechanism; and cohort-dependent effects erode performance during validation [3,10,20]. These vulnerabilities are not idiosyncratic or technical edge cases—they are intrinsic to the measurement of metabolites as dynamic, context-sensitive reporters of physiology. Treating such signals as static, disease-specific indicators inevitably produces fragile biomarkers that fail under real-world clinical conditions.

A central message of this review is that clinical translation requires a shift in perspective. Rather than asking whether a metabolite is statistically associated with disease, investigators must ask whether the measurement is robust, interpretable, and decision-relevant under clinically realistic conditions. This reframing moves biomarker development away from post hoc rationalization of discovery-phase findings and toward disciplined, stage-specific evaluation aligned with clinical decision-making. The decision-gate framework, summarized in Table 1 and mapped onto the translational pipeline in Figure 1, provides a practical structure for implementing this shift.

Importantly, abandoning the pursuit of single-metabolite biomarkers does not imply abandoning metabolomics or lipidomics as clinical tools. On the contrary, these platforms may be most powerful when used to inform composite, context-aware models rather than isolated decision rules. Multi-metabolite panels, pattern-based signatures, and mechanism-informed interpretations that reflect pathway activity or metabolic flux offer more realistic paths to clinical utility [16,43]. When developed within transparent, rigorously validated pipelines, such approaches can harness the strengths of metabolomics while mitigating its inherent vulnerabilities [27,71].

Looking forward, progress will depend on tighter integration between discovery scientists, clinicians, laboratory professionals, and regulatory stakeholders. Early alignment with intended clinical use, explicit acknowledgment of analytical and biological limitations, and adherence to emerging reporting and validation standards are essential [21,27]. Biomarkers that cannot withstand routine clinical variability, lack molecular definition, or fail to demonstrate stable performance across cohorts should be deprioritized early—before substantial resources are committed to validation or commercialization.

Ultimately, the goal of metabolomics and lipidomics in medicine is not to maximize the number of statistically significant features reported but to generate measurements that meaningfully improve clinical decision-making. By treating metabolites as components of a translational system rather than isolated discovery signals, the field can move beyond recurrent cycles of promise and disappointment toward durable clinical impact. The roadmap presented here is intended not as a restrictive checklist but as a pragmatic guide for transforming metabolic measurements into decision-grade biomarkers that can survive the demands of real-world clinical practice. The proposed framework is conceptual and does not substitute for disease-specific validation studies. Quantitative cutoffs remain context-dependent, and the model may not fully capture emerging modalities such as spatial metabolomics or metabolic imaging.

## Figures and Tables

**Table 1:** Translational failure modes and decision gates for metabolic biomarkers.

Translational Stage	Dominant Failure Mode	Key Diagnostic Questions	Decision-Gate Implication
Pre-analytical handling	Sensitivity to fasting status, circadian rhythm, handling conditions, and storage instability	• Is the biological compartment appropriate for the intended clinical use? • Are metabolite levels stable under realistic variation in fasting, timing, and routine handling? • Is the analyte resistant to degradation, oxidation, or enzymatic remodeling during collection and storage?	No → Discontinue as a standalone biomarker. Pre-analytical fragility represents a hard constraint that cannot be corrected downstream by normalization or modeling.
Analytical validity	Ionization bias, matrix effects, batch dependence, semi-quantitative measurement	• Does signal intensity reflect molar abundance or ionization efficiency? • Can internal standards adequately correct analyte-specific variability? • Is performance stable across batches, platforms, and laboratories?	No → Restrict to exploratory or research use. Do not advance to decision-grade claims or fixed clinical thresholds.
Structural and annotation confidence	Ambiguous molecular identity, unresolved isomers, analytical artifacts	• Is the analyte unambiguously identified at the molecular level? • Are unresolved isomers or in-source artifacts likely contributors to the signal? • Is annotation confidence reported transparently?	No → Do not advance as a single-analyte biomarker. Consider incorporation into multivariate or pathway-level models only.
Biological interpretability	Dominance of systemic stress responses, lack of disease specificity	• Does the metabolite report disease-specific mechanisms or generalized physiological stress? • Is interpretation consistent with known metabolism and physiology? • Are alternative explanations (inflammation, hypoxia, medication effects) excluded?	No → Limit mechanistic and clinical claims. May contribute to pattern-based signatures but lacks standalone clinical meaning.
Statistical robustness	Multiple testing, overfitting, optimism bias	• Was feature selection separated from performance evaluation? • Is performance stable under external or prospective validation? • Are negative or null results transparently reported?	No → Re-evaluate model or feature set. Statistical significance alone is insufficient for translational advancement.
Cohort validation	Cohort shift, hidden confounders, threshold instability	• Does performance persist across heterogeneous populations? • Are decision thresholds stable across cohorts? • Is recalibration required for each new setting?	No → Incompatible with routine clinical use. Biomarkers requiring cohort-specific tuning lack regulatory viability.
Clinical utility	Absence of actionable impact on decision-making	• Does the biomarker improve diagnosis, prognosis, or treatment decisions beyond existing standards? • Is the intended use case clearly defined? • Are downstream clinical consequences specified?	No → Do not pursue clinical deployment. Analytical and biological validity without utility does not justify implementation.

## Data Availability

No new data were created or analyzed in this study. Data sharing is not applicable to this article.

## Abbreviations

**AUC**	Area Under the Curve
**BEST**	Biomarkers, EndpointS, and other Tools
**EMA**	European Medicines Agency
**ESI**	Electrospray Ionization
**FDA**	Food and Drug Administration
**IVDR**	In Vitro Diagnostic Regulation
**MSI**	Metabolomics Standards Initiative
**QC**	Quality Control
